# Voluntary exercise in mice triggers an anti-osteogenic and pro-tenogenic response in the ankle joint without affecting long bones

**DOI:** 10.1016/j.bonr.2024.101810

**Published:** 2024-10-15

**Authors:** Anne Briolay, François Duboeuf, Séverine Delplace, Leyre Brizuela, Olivier Peyruchaud, David Magne, Carole Bougault

**Affiliations:** a Universite Claude Bernard Lyon 1, CNRS, UMR 5246, ICBMS, F-69622 Villeurbanne, France; b Universite Claude Bernard Lyon 1, INSERM, UMR 1033, LYOS, F-69372 Lyon, France; c Universite Littoral-Côte d'Opale, ULR 4490, MABLab, F-62327 Boulogne/Mer, France

**Keywords:** Voluntary exercise, Ankle joint, Tendon, Bone, Ossification

## Abstract

Biomechanical stimulation is proposed to occupy a central place in joint homeostasis, but the precise contribution of exercise remains elusive. We aimed to characterize in vivo the impact of mechanical stimulation on the cell-controlled regulation of ossification within the ankles of healthy mice undergoing mild physical activity. DBA/1 male mice were subjected to voluntary running exercise for two weeks, and compared to mice housed in standard conditions (*n* = 20 per group). Free access to activity wheels resulted in a running exercise of 5.5 ± 0.8 km/day at 14.5 ± 0.5 m/min. Serum levels of alkaline phosphatase, IL-6, IL-8/Kc, IL-17a, and TNF-α were measured. No change in systemic inflammation was detected. The bone architecture of the femur and the calcaneus was unchanged, as revealed by μCT and histology of the enthesis of the Achilles tendon. mRNAs were extracted from femurs, tibias, and ankle joints before RT-qPCR analysis. The expression of the mechanosensitive genes *Sclerostin* (*Sost*) and *Periostin* (*Postn*) was not impacted by the exercise in long bones. Oppositely, *Sost* and *Postn* levels were modulated by exercise in joints, and osteogenic markers (*Col10a1*, *Runx2*, *Osx*, and *Dmp1*) were downregulated in the exercise group. In addition, the tenogenic markers *Scx*, *Mkx*, and *Tnmd* were upregulated by exercise. Thus, voluntary exercise affected the phenotype of joint cells without impacting long bones. As gene expression of *Bmp2*, *Bmp4*, and *Id1* was also reduced in these cells, an off-regulation of BMP signaling could be partly responsible for their mechanosensitive response. Running exercise seemed to preserve the tendon from its progressive ossification, as seen in numerous enthesopathies. This study paves the way to future experiments for investigating the effects of mechanical stimulation in various mouse models.

## Highlights

• Access to the activity wheel led to a homogeneous, physiological running exercise.
• A mechanosensitive response was detected in the joints, but not in the long bones.
• The bone architecture of the femur and calcaneus was not altered by the exercise.
• Exercise downregulated osteogenic markers and BMP signaling in joint samples.
• Tenogenic markers *Scx*, *Mkx*, and *Tnmd* were upregulated by exercise.

## Graphical abstract

**Figure:**
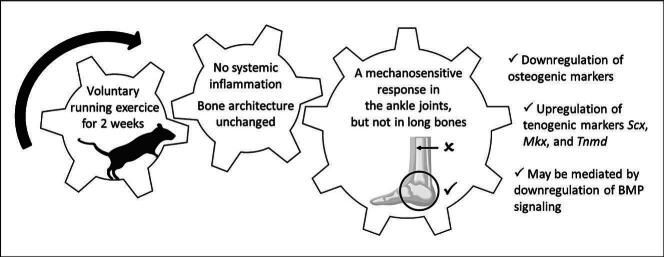

## 1 Introduction

Obviously, physical activities impact the whole musculoskeletal system. Mechanical stimulation modifies the mass, structure, and quality of the bone, as it accommodates a changing environment. The regulation of bone formation and resorption occurring during exercise follows rules known since the 90s as “mechanostat” (Hughes and Petit, 2010). Mechanical stimulation is also proposed to occupy a central place in joint homeostasis. In agreement, physical therapy and exercise are recommended as non-pharmacologic therapy in almost all joint disorders. On the other hand, biomechanical factors also contribute to various enthesopathies, since the entheses are sites of high mechanical strain at the muscle-tendon-bone junction (Benjamin et al., 2006). In osteoarthritis (OA), which is the most frequent joint disorder, altered loading is a known risk factor for cartilage degeneration (Guilak, 2011). Concerning spondyloarthropathies (SpA), which are the third most common rheumatic disorder after rheumatoid arthritis and OA, the consensual hypothesis is that local mechanical stresses in the enthesis are generated by inflammation-induced bone architecture disorganization and that, in turn, they exacerbate local inflammation and promote new bone formation. Indeed, ectopic joint ossification may arise at the late stages of the disease, in the form of bone spurs called enthesophytes. Physical activity is recommended in SpA, since exercise, such as energetic walking or swimming, improves physical performance, in particular with cardiovascular and flexibility benefits (Fabre et al., 2016). Mechanical strain therefore also stands in a crucial position in the pathophysiology of SpA (Neerinckx and Lories, 2017; Jacques and McGonagle, 2014; Jacques et al., 2013). The contribution of the mechanical component to joint homeostasis most likely involves the mechanosensitive character of multiple tissues, including tendon, synovium, cartilage and bone. An extensive study on various arthritis mouse models recently established that mechanical strain is crucial for the site-specific localization of inflammation and tissue damage (Cambré et al., 2018). However, the precise molecular and cellular mechanisms underlying the pathological response of the joint tissues remain elusive, particularly regarding the control of ossification process.

We aimed to characterize in vivo the impact of mechanical stimulation on the entheses of a bearing joint in mice. As a first approach, we focused on healthy mice subjected to mild physical activity. Among the various exercise models (isolated loading, forced treadmill running, swimming, resistance training, unloading, and vibration modes), we selected the voluntary wheel running, whose main advantages are its physiological relevance and putative translatability (Portier et al., 2020). Cambré et al. demonstrated the impact of enhanced activity on arthritis progression by the use of a similar protocol of 2-week long voluntary running (Cambré et al., 2018). We have chosen the DBA/1 mouse model, which has previously allowed demonstrating the mechanosensitive facet of the SpA disease (Jacques et al., 2013). In addition, DBA/1 male mice can develop arthritis spontaneously, with enthesophytes and ankylosis, mainly affecting the proximal interphalangeal joints and the ankle of the hind limbs, and comparable to human SpA features (Braem et al., 2012; Nordling et al., 1992). We focused on the ankles, because they are the weight bearing joints the most exposed to mechanical loading (Chang et al., 2016), and because among many entheses, they contain the insertion of the Achilles tendon into the calcaneus bone, which is the archetype of the fibrocartilaginous enthesis (Benjamin et al., 2000) and one of the zones subjected to ectopic ossification in SpA patients (Lories and Schett, 2012). Long bones of the hind paw were used for comparison. We dedicated the molecular analysis to investigate the effect of exercising on cell differentiation. Gene markers of tendon, cartilage, and bone tissue were targeted with the objective to detect any modification of cell phenotype that could contribute to the ossification regulation. We also examined some players of the bone morphogenetic protein (BMP) pathway, known to play an essential role in the process of ectopic bone formation (Kan et al., 2018). Formation of bony spurs may result of increased osteoblastic activity, or of process resembling endochondral ossification (Benjamin et al., 2000), with the reorientation of fibro-chondrocytes or the metaplasia of tendon cells into hypertrophic chondrocytes or osteoblast-like cells (Aghajanian and Mohan, 2018; Magne and Bougault, 2015).

## 2 Methods

### 2.1 Animals and voluntary wheel-running exercise

All procedures were in accordance with the European directives and the protocols were approved by the French and European ethics committees for animal use and care (APAFIS#24732-2020031916053655). Male DBA/1 mice were purchased from Janvier labs at the age of 11 weeks and separated into two groups: exercise (*n* = 20) and control (Ctrl; n = 20). All animals were supplied with food and water ad libitum, housed individually, on a 12:12 h dark–light cycle (7 AM–7 PM) and allowed to adapt to their new environment for 1 week. To study the effect of voluntary wheel-running exercise, animals were housed in commercial cages (Bioseb) with a spontaneous activity wheel (23 cm in diameter). They had free access to the wheel for 2 weeks. The embedded electronics measured wheel revolutions, speed, and utilization time continuously (ACTIVW-SOFT software, Bioseb). Control animals were housed in standard conditions. Mice were weighed weekly.

### 2.2 Serum analysis

Serum alkaline phosphatase (ALP) specific enzymatic activity was measured using *p*-nitrophenyl phosphate (Sigma-Aldrich) as substrate and a kinetic measurement of the absorbance at 405 nm as described before (El Jamal et al., 2019). Serum concentrations of IL-6, IL-8/Kc, IL-17a, and TNF-α were measured by Luminex technology using a mouse premixed multi-analyte kit (R&D system, Biotechne) in accordance with the manufacturer's instructions. In brief, 50 μL of serum or standard were loaded in duplicates. Samples were successively incubated with antibody-linked microparticles (2 h), with biotinylated secondary antibodies (1 h), and with streptavidin-fluorophore conjugate (30 min), before reading the plate by the use of a Luminex Magpix (Luminex Corporation). The software automatically generated standard curves.

### 2.3 Microcomputed tomography analysis (micro-CT) and Histology

After fixation in ethanol, the distal femur and calcaneus from 11 or 12 mice per group were analyzed by X-ray microcomputed tomography (micro-CT), by the use of the Skyscan 1176 scanner with a voxel size of 9.08 μm (Bruker Biospin). Radiographic settings, image reconstruction, and three-dimensional modeling and analysis were executed as previously described (Flammier et al., 2019). For long bone histomorphometry, the volume of interest (VOI) was a cylinder 1 mm in height, either at the femoral midshaft for cortical analysis or at the distal femoral metaphysis for trabecular analysis. For calcaneus exploration, the entire bone was considered as VOI. The ankles of the other mice were fixed for various histologic analysis. Mineralized-matrix staining using Von Kossa dye was performed on some sections as previously published (Lencel et al., 2011). Others were decalcified prior to paraffin embedment and either stained using Hematoxylin-eosin-saffron (HES) to investigate general morphology (performed in the Novotec lab, Bron, France) (El Jamal et al., 2019) (Saffron, which colors collagen fibers, is used to help differentiate between muscle, stained red or pink, and connective tissue, stained bright yellow or orange) or stained using Safranin O/Fast Green (performed in the ImaFlow corefacility, US58 BioSanD, Inserm, University of Burgundy, Dijon, France) for the detection of cartilage (stained red, the background is stained bluish green).

### 2.4 Immunohistochemistry (IHC)

IHC experiments were carried out in the ImaFlow corefacility, US58 BioSanD, Inserm, University of Burgundy, Dijon, France. The samples (*n* = 3 per group) were fixed in 4 % paraformaldehyde for 48 h, decalcified in 10 % EDTA solution for 7 days, then fixed again for 24 h. After dehydration and embedding in paraffin, 5 μm thick slices were made on a rotary microtome and deposited on coated glass slides. For the antigen retrieving of Sost, rehydrated tissue sections were incubated in a proteinase K solution at pH 8 at 37 °C for 20 min. For the antigen retrieving of Tnmd and Pro-Bmp2, rehydrated tissue sections were incubated in a 0.5 % Triton-X100 solution at room temperature for 4 min. Next, tissue sections were incubated in 3 % hydrogen peroxide for 15 min and in 3 % bovine serum albumin for 20 min. Sections were then incubated with the primary antibody overnight at 4 °C (Goat anti SOST, R&D Systems, #AF1589; Rabbit anti ALPL, Novus Biologicals, #NBP2-67295; Rabbit anti TNMD, Fisher Scientific, #17238803; Mouse anti pro-BMP2, Novus Biologicals, #MAB2260), and with a secondary antibody conjugated with polymerase-HRP (Vector Laboratories) for 45 min at room temperature. Chromogenic revelation was performed using NovaRed kit (Vector Laboratories). Tissue sections were then counterstained with Harris Hematoxylin (Leica). After dehydration, tissue sections were mounted with organic mounting medium (Leica). Images of stained sections were realized on an AxioImager M2 microscope (Zeiss) equipped with an Axiocam 305 (Zeiss) camera.

### 2.5 Gene expression analysis

Hind paws were retrieved from all mice, and dissected for skin and muscles removal (n). For the thereafter-called “long bones” samples, the femur and tibia were flushed and pooled together. For the “joint” samples, a piece of 10–15 mm^3^ was isolated within the ankle, including the enthesis of the Achilles tendon at the dorsal end of the calcaneus (Suppl. Fig. 1). Our “joint” tissue samples should thus contain trabecular, cortical, and subchondral bones, tendons and ligaments, growth plate cartilages, and fibrocartilages of the entheseal, the sesamoid, and the periosteal regions (Tits et al., 2021). Tissue samples were stocked at −80 °C before grinding in a liquid nitrogen-cooled mini mortar. Total RNA isolation was performed using the TRIzol reagent method (Sigma-Aldrich) combined with silica-based membrane columns: the aqueous phase obtained after TRIzol/chloroform separation was completed to 35 % ethanol and loaded on a Nucleospin column (Macherey-Nagel). The sample was then purified using an on-column DNase treatment, according to the manufacturer's standard protocol. Reverse transcription (RT) and real-time PCR were performed as previously described (El Jamal et al., 2019), with *Actb*, *Hprt*, and *Rpl13a* as reference genes. Relative gene expression was calculated using the comparative Ct (cycle threshold) method quantification (2^-ΔCt^). The qPCR work was performed using the facilities of the DTAMB platform (FR BioEEnViS, Université Claude Bernard Lyon 1).

### 2.6 Statistical analysis

Data are expressed as mean ± standard deviation (SD) or ±95 % confidence interval (CI). Statistical analysis was performed with the two-sided Mann-Whitney tests to compare mean values between 2 groups, using GraphPad Prism software (GraphPad). Statistical significance was defined as *p* < 0.05.

## 3 Results

### 3.1 Characterization of the wheel-running exercise

**Fig. 1:**
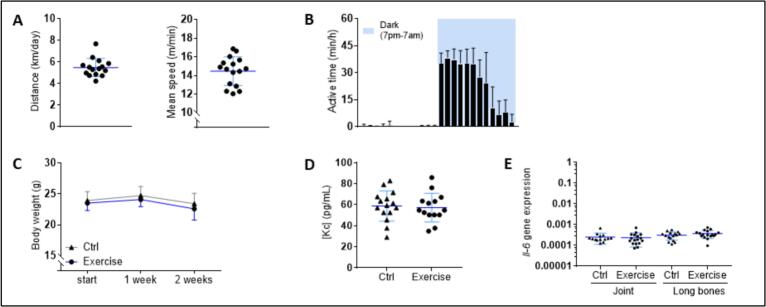
Characterization of the 2-week wheel-running exercise with DBA/1 mice. (A–B) The running performances of each mouse of the exercise group was continuously registered, allowing the calculation of the daily distance and mean speed reached (A) and the assessment of the hourly distribution of activity times over a day (data from a single mouse, followed over 14 days) (B). (C) The exercise group was compared to the control group, housed in standard conditions. No difference was observed regarding the evolution of the body weight over the 2-week protocol. (D–E) At the end of the experiment, the serum concentration of IL-8/Kc (D) and the relative gene expression of *Il6* (E) in tissue extracts were similar in both groups. Data were obtained from *n* = 14 to *n* = 17 mice per group, and represented as the mean with 95 % confidence interval (CI).

The running performances were recorded throughout the 2-week protocol. Free access to the activity wheel resulted in a voluntary running exercise of 5.5 ± 0.8 km/day (approximately 80 km in total) at the average speed of 14.5 ± 1.5 m/min (Fig. 1A). As expected, the activity periods were restricted to dark hours (Fig. 1B). The body weight did not vary over the two-week period and no difference was noticed between the control and the exercise group (Fig. 1C). Serum samples from both groups were analyzed by Luminex assay for cytokine detection. No systemic inflammatory response was detected as IL-6, IL-17a, and TNF-α were not detected (i.e. concentrations were lower than 8, 14 and 1 pg/mL, respectively). IL-8/Kc serum levels were similar in the control and the exercise groups (59 ± 14 vs 57 ± 14 pg/mL, Fig. 1D). *Il6* gene expression was very faint and constant in all tissue extracts, suggesting that exercise impacted neither joint nor bone inflammation (Fig. 1E). No sign of inflammatory infiltration was detected in HES-stained tissue sections (data not shown).

### 3.2 Mechanosensitive regulation of bone markers in the joint, but not in long bones

**Fig. 2:**
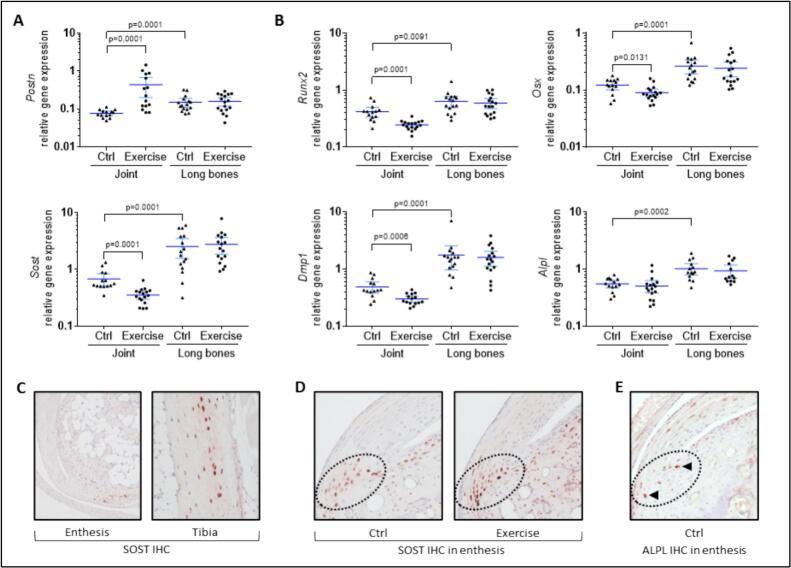
Regulation of bone markers in response to exercise and comparison between joint and long bone samples. (A–B) After RNA extraction from tissue samples, relative gene expression was measured by RT-qPCR for the mechanosensors *Sclerostin* (*Sost*) and *Periostin* (*Postn*) (A) and for the bone formation markers *Osterix* (*Osx*), *Runx2*, *Dentin matrix acidic phosphoprotein* (*Dmp1*), and *Tissue non-specific alkaline phosphatase* (*Alpl*) (B). Data were obtained from *n* = 14 to *n* = 17 mice and represented as the mean with 95 % confidence interval (CI). (C–D) Immunolocalization of SOST expression by IHC (representative picture of *n* = 3 per group). (C) Positive staining of osteocytes in bone structures of control samples either surrounding the enthesis or in tibia cortical zone, (D) Focus on the enthesis, where chondrocyte-like cells at the Achilles tendon insertion site in the calcaneus were also positive (dotted circle), and comparison between the exercise and the control groups. (E) Immunolocalization of ALPL expression by IHC, focus on the enthesis (dotted circle), where few chondrocyte-like cells were positive (arrowheads).

First, *Sclerostin* (*Sost*) and *Periostin* (*Postn*) gene expression was monitored as bone mechanosensitive response. Both are known to be involved in bone response to loading; the first event is certainly *Postn* overexpression, which leads in a second time to *Sost* inhibition (Bonnet et al., 2009; Gerbaix et al., 2015). In our model, their gene expression was indeed drastically modified in the exercise group compared to the controls, but only in joint tissues (*p* < 0.001), as no modulation was observed in long bones (Fig. 2A). The expression of *Postn* was increased 5 folds while *Sost* mRNA was reduced by half (Fig. 2A). Meanwhile, expression of the bone formation master transcription factors *Osterix* (*Osx*) and *Runx2* was downregulated by wheel-running exercise, only in the joint samples (Fig. 2B, −26 % and −41 %, respectively). Overall bone homeostasis was thus affected in joint samples, with *Runx2* involved in early osteoblast differentiation and *Osx* in later osteoblast maturation and further differentiation into osteocytes (Chan et al., 2021). On the one hand, *Sost* is a transcript specific to terminally differentiated osteocytes, which are deeply embedded in the bone matrix, and on the other, *Postn* is a selective marker of periosteal cells, which cover the outer surface of bone (Nookaew et al., 2024). This tissue-specific pattern of the exercise-induced downregulation was also verified for *Dentin matrix acidic phosphoprotein* (*Dmp1*) mRNA. *Tissue non-specific alkaline phosphatase* (*Alpl*) gene expression was unchanged (Fig. 2B). As anticipated, all bone markers were substantially more expressed in long bone samples than in joint samples (Fig. 2B, *p* < 0.001 for all genes). Consistently, SOST protein was detected in osteocytes embedded in the bone matrix by IHC (Fig. 2C). However, focusing on the enthesis, some chondrocyte-like cells were also positive at the Achilles tendon insertion site in the calcaneus (Fig. 2D). No difference of expression pattern or staining intensity was noticeable between the exercise group and the control samples (Fig. 2D). Therefore, the exercise-induced downregulation of *Sost* detected by RT-qPCR in joint tissues may not reveal extinction in any particular cell population, but rather a global decrease in gene expression in all *Sost*-expressing cells. The presence of ALPL at protein level in the enthesis was confirmed by IHC, as a small number of chondrocytes at the mineralization front were positively labelled (Fig. 2E). Consistent with the *Alpl* gene expression results, no differences were detected between the exercise group and control samples.

### 3.3 Absence of exercise-induced modification in bone structures

**Fig. 3:**
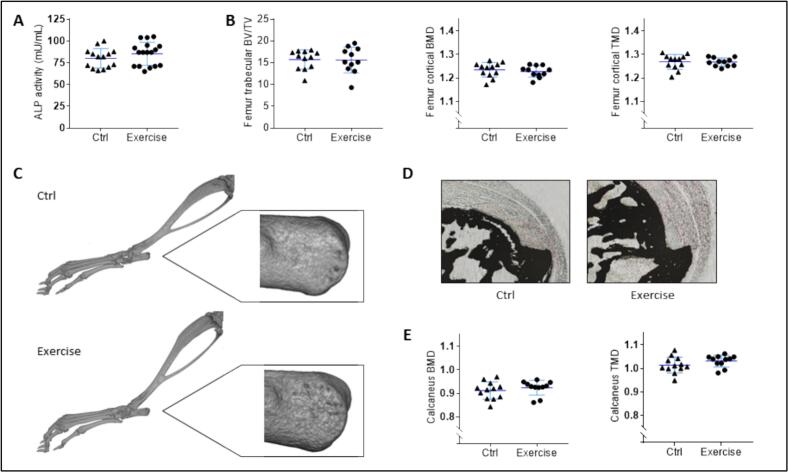
Analysis of bone structure. At the end of the 2-week wheel-running protocol, no modification of bone structure parameters was detected between the exercise and control groups. (A) Serum levels of alkaline phosphatase (ALP) activity were assessed as a marker of bone formation (*n* = 14 or 17). (B–C) Bone architecture was analyzed by μCT. For the femur, bone volume fraction (BV/TV) and bone and tissue mineral density (BMD and TMD) were quantified (B) (*n* = 11 or 12). The morphology of the ankle joint and in particular the calcaneal tuberosity (zoom insert) was carefully observed (C). (D) Demarcation of mineralization of the Achilles tendon enthesis was visualized by von Kossa staining (*n* = 4), and (E) the BMD and TMD of the calcaneus were measured by μCT (n = 11 or 12).

Serum ALP activity, which is a typical marker of bone formation, was assessed at the end of the experiment. No exercise-induced variation was detected (Fig. 3A). Structural parameters of the femurs were measured by μCT: bone volume fraction (BV/TV) of the trabecular zone and bone and tissue mineral density (BMD and TMD) of the cortical zone were unmodified by the wheel-running exercise (Fig. 3B). We then examined the ankle joint (Fig. 3C), focusing on calcaneus and the enthesis of the Achilles tendon. No morphological changes were observed at the tendon's bony anchoring site, at the calcaneal tuberosity (Fig. 3C). Von Kossa staining was performed on 4 samples of each group to visualize the mineralization demarcation. No difference was visible between the samples (Fig. 3D). As in the femur, μCT measurement in the calcaneus demonstrated that bone and tissue mineral densities were unchanged by the exercise (Fig. 3E).

### 3.4 Exercise-induced regulation of cartilage and tendon markers in the joint

**Fig. 4:**
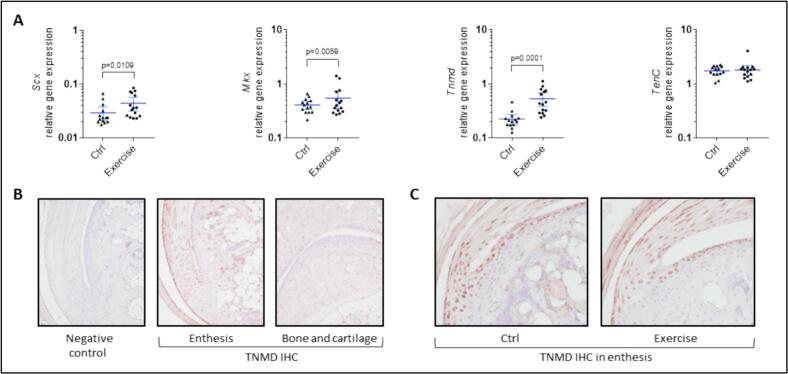
Regulation of tendon markers in the joint in response to exercise. (A) Relative gene expression for the tenogenic markers *Scleraxis* (*Scx*), *Mohawk homeobox* (*Mkx*), *Tenomodulin* (*Tnmd*), and *Tenascin C* (*TenC*) by RT-qPCR. Data were obtained from *n* = 14 to *n* = 17 mice and represented as the mean with 95 % confidence interval (CI). (B–C) Immunolocalization of TNMD expression by IHC (representative picture of *n* = 3 per group). (B) Positive staining of tenocytes within the Achilles tendon, compared to the absence of expression in bone and articular cartilage tissues. (C) Focus on the enthesis, where no difference was noticed between the exercise and the control groups.

We then investigated the effect of the wheel-running exercise on tendon and cartilage differentiation markers within the ankle joint. Tenogenic differentiation was stimulated in the exercise group, as revealed by the upregulation of the master transcription factors *Scleraxis* (*Scx*) and *Mohawk homeobox* (*Mkx*) (Fig. 4A, approximately 1.5 fold, *p* < 0.02). In accordance, the transmembrane glycoprotein *Tenomodulin* (*Tnmd*), which is typically expressed at high levels in tenocytes and ligamentocytes, was increased (Fig. 4A, 2.4 fold, *p* < 0.001). Besides, the expression of the *Tenascin C* (*TenC*), which is more prevalent in the spherical-shaped tendon cells of the fibrocartilagenous regions (De Palma et al., 2004; Martin et al., 2003), remained unchanged (Fig. 4A). The restriction of TNMD expression to the tenocyte cell-type was confirmed by IHC; no expression was detected neither in bone nor in superficial articular cartilage. Focusing on the enthesis, no difference of localization was observed between the exercise and the control groups (Fig. 4B).

**Fig. 5:**
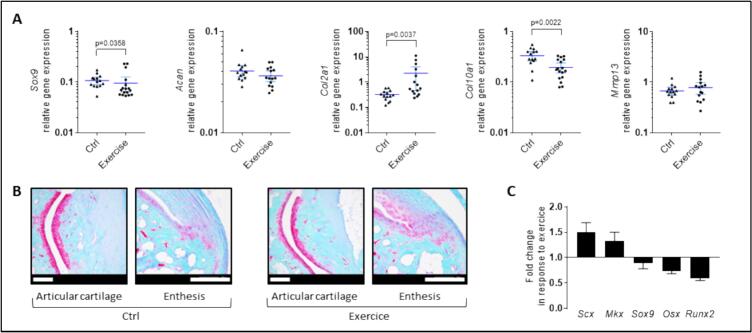
Regulation of cartilage markers in the joint in response to exercise. (A) Relative gene expression was measured by RT-qPCR for some cartilage markers, either for proliferative chondrocytes (*Sox9*), *Aggrecan* (*Acan*), and *Type II collagen* (*Col2a1*), or for hypertrophic chondrocytes (*Type X collagen* (*Col10a1*) *or Matrix Metallopeptidase 13* (*Mmp13*)). Data were obtained from *n* = 14 to *n* = 17 mice and represented as the mean with 95 % confidence interval (CI). (B) Safranin O/fast green staining was performed on ankle tissue sections to visualize (in pink) superficial articular cartilages (left panel) or fibrocartilaginous zones of the enthesis (right panel) (representative picture of *n* = 3 per group; scale bars = 200 μm). (C) Alternative plot of the fold change in relative gene expression in response to exercise of *Scx*, *Mkx*, *Sox9*, *Osx*, and *Runx2*, represented as the Exercise/Control ratio (mean with SD).

In parallel, the chondrogenic transcription factor *Sox9* and the chondrocyte marker *Aggrecan* (*Acan*) seemed negligibly affected by the exercise (Fig. 5A, around −10 %). However, the expression of *Col2a1* mRNA encoding Type II collagen, which is the major cartilage-matrix component, was drastically increased in the exercising group (7-fold, *p* < 0.01). Both aggrecan and type II collagen are typically present in growth plate and articular cartilage as well as in fibrocartilage of the entheseal, sesamoid and periosteal regions (Waggett et al., 1998). Safranin O staining for glycosaminoglycans did not reveal any obvious difference between the exercise and the control group, neither in superficial articular cartilages nor in the fibrocartilaginous zone of the enthesis (Fig. 5B). Regarding the indicators of hypertrophic chondrocytes, much like the osteogenic markers, they were either downregulated in the wheel-running group (*Col10a1* encoding Type X collagen) or unchanged (*Mmp13* encoding Matrix Metallopeptidase 13) (Fig. 5A). Globally, the RT-qPCR analysis suggested an anti-osteogenic and pro-tenogenic response in the ankle joint, as recapitulated Fig. 5C, focusing on the response to exercise of the 5 key transcription factors analyzed.

### 3.5 Involvement of the BMP pathway

**Fig. 6:**
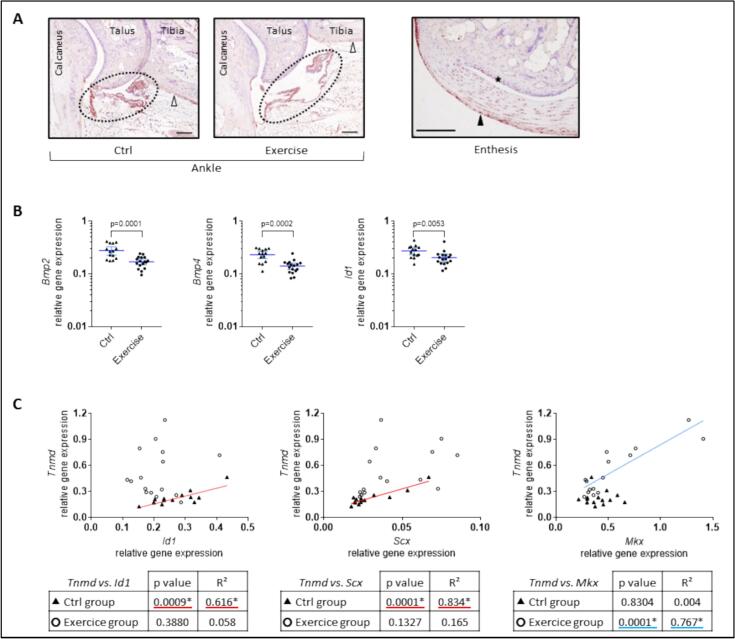
Involvement of BMP (Bone Morphogenetic Protein) signaling in the pro-tenogenic response to exercise. (A) BMP-2 production was detected by pro-BMP-2 immunostaining in the ankle joint (representative picture of *n* = 3 per group). Its expression was localized in the synovial membrane (dotted circle), in the periosteal region of the tibia (open arrowhead), in the tendon (right panel) and even more so in its epitenon (black arrowhead), and in the sesamoid fibrocartilage (star). Scale bars = 200 μm. (B) Relative gene expression was measured by real-time RT-qPCR for the *Bmp2* and *Bmp4*, and the BMP-target gene *Inhibitor of DNA binding* (*Id*) *1*. Data were obtained from *n* = 14 to *n* = 17 mice and represented as the mean with 95 % confidence interval (CI). (C) Pearson correlation coefficients were calculated to relate *Tnmd* gene expression to *Mkx*, *Scx* or *Id1* mRNA levels (*p < 0.001 and R^2^ > 0.6). RT-qPCR data from each mice (*n* = 29) were plotted to illustrate these correlations. Samples from the control group are represented by black triangles and samples from the exercise group by open circles. Linear regressions calculated from control samples are plotted in red; those from exercise samples are plotted in blue.

Pro-BMP-2 immunostaining was used to localize BMP-2 production in the ankle joint. The synovial membrane and periosteum of the tibia showed positive staining, perhaps slightly less marked in the exercise group (Fig. 6A, left panel). Pro-BMP-2 expression was also detected in the tendon and its surrounding epitenon, as well as in sesamoid fibrocartilage, with no difference between the two groups (Fig. 6A, right panel). A decrease in *Bmp2* and *Bmp4* gene expression was induced by exercise (Fig. 6B). In parallel to *Bmp2* and *Bmp4* downregulation, we noticed a decrease in *Id1* encoding Inhibitor of DNA binding 1, a well-known target gene of BMP signaling that involves the Smad activation cascade (Lewis and Prywes, 2013) (Fig. 6B). Therefore, we suggest that a downregulation of the BMP signaling may mediate the anti-osteogenic and pro-tenogenic orientation of the joint cells in response to exercise.

Id proteins are transcriptional regulators that act by inhibiting the function of other basic helix-loop-helix (bHLH) transcription factors. Remarkably, the expression of the tenogenic marker *Tnmd* strongly correlated with *Id1* expression only in the control group, and not in the exercise group (*p* = 0.0009 vs *p* = 0.3880; Fig. 6C). In physiological conditions, *Tnmd* is typically activated downstream Scx (Shukunami et al., 2018), which is a bHLH transcription factor. Again, the correlation between *Tnmd* gene expression and *Scx* mRNA levels was also much stronger in the control group than in the exercise group (*p* = 0.0001 vs *p* = 0.1327; Fig. 6C). Along with Scx, the homeobox protein Mkx has been identified as a crucial transcription factor in tendon development and differentiation (Liu et al., 2014). On the contrary, a correlation between *Tnmd* and *Mkx* was detected in the exercise group (p = 0.0001, R^2^ = 0.767; Fig. 6C) but not in the control group. These data suggest that in mechanically stimulated tenocytes, *Tnmd* expression is regulated by the typical tenogenic transcription factor Mkx whereas in the absence of exercise, the activation of BMP signaling would lead to Id1- and bHLH-mediated regulation of *Tnmd*.

## 4 Discussion

Our experimental design revealed that a mild mechanical stimulation triggers a response after only 2 weeks of practice in the ankles of mice. The voluntary wheel running allowed a not stressful and physiological exercise that respected the natural dark and discontinuous activity of the animals. Spontaneous running clearly affects the total physical activity of mice (Silvennoinen et al., 2014), even if the physical effort overload, relative to standard-housed mice, is rather small in that model. Our procedure resulted in a rather homogeneous activity with <5 % of variation for the mean speed and <15 % for the daily distance. The short duration of our protocol especially allowed us to bypass the potential variations due to the weight or age of the animals (Portier et al., 2020). A known limitation of the voluntary wheel running model is the high variability between the different mouse strains; the DBA/1 being one of the less performing mice (Lerman et al., 2002). Performances may also depend on the age and the sex of the animals. In addition, caution should be taken when interpreting the running parameters, as in particular, the design of the wheels (i.e. diameter, resistance…) affects the performances (Seward et al., 2018). Nevertheless, our mice ran a creditable average for a 2-week long protocol of 5.5 ± 0.8 km/day (Cambré et al., 2018; Zhang et al., 2017).

Our first main result is the absence of exercise-induced modification in long bones, regarding either the bone structure or the mechanosensitive gene response. A recent review revealed that only 62 % of the studies using voluntary wheel running with rats showed a change in bone quality (Portier et al., 2020). In mouse, it seems that longer protocols are needed to detect any modification of long bone structure by μCT. In C57Bl/6 at least, free access to the wheel during 5 months appeared to induce bone structural adaptations, while 1 month of exercise is not enough (Isaksson et al., 2009; Ma et al., 2010; Schlecht et al., 2018). However, Zhang et al. have shown that a voluntary exercise for 2 weeks increased some bone gene markers, including *Osx*, in femur and tibia of C57Bl/6 mice (Zhang et al., 2017). Again, the exercise-induced bone remodeling is certainly highly strain-dependent (Schlecht et al., 2018).

Our second main result came from the analysis of ankle joint samples. As opposed to our results in long bones, the mechanosensors were neatly activated in the joints of the exercising group, as evidenced by the rise in *Postn* expression, correlating with a drop in *Sost* expression (Bonnet et al., 2009; Gerbaix et al., 2015). Entheses and bone-to-tendon interfaces are very sensitive to exercise, so that little mechanical stimulation seems sufficient to enhance their biomechanical properties. For example, a low intensity exercise, at the adequate post-operative timing, can facilitate rotator cuff repair in mice (Chen et al., 2021; Lu et al., 2021). In the same vein, selecting mice upon their preference for exercise is enough to observe an enlargement of the humerus deltoid tuberosities, while the femoral trochanter remains unchanged (Castro et al., 2021). It is likely that the bearing joints, which contain many enthesis sites, react earlier than long bones to any mechanical stimulation of the paw. Other experiments should be conducted at various time points, either to detect an even earlier response in the joint or to reveal how long bones react with longer stimulations.

Third, we further looked for any gene expression modulation that could contribute to the cell-controlled regulation of the ossification within the joint, even if the structure of the calcaneus and especially its posterior end, seemed unaltered. In the bone field, the mechano-regulation of *Postn* and *Sost* is usually accompanied by a stimulation of bone formation (Gerbaix et al., 2015; Sapir-Koren and Livshits, 2014). In our model however, the mechanical stimulation led to an unexpected anti-osteogenic response within the ankles, with an exercise-induced downregulation of *Col10a1*, *Runx2*, *Osx* and *Dmp1*. Admittedly, we analyzed singular bony regions in our samples (Tits et al., 2021), compared to the typical studies focusing on load-bearing long bones. We concluded that our 2-week-long protocol did not trigger osteoblastogenesis or increase osteoblastic activity. Nevertheless, our results concerning the bone remodeling should be considered in the light of some limitations, as we did not explore the catabolic aspect and the associated osteoclastogenic markers, in particular. We next wondered whether exercise onsets a process resembling endochondral ossification, with a reorientation of tendon cells into chondrocyte-like cells. Especially because in the SpA model of aging DBA/1 male mice, enthesis progenitors seem to undergo chondrogenic differentiation at the beginning of the ankylosis process (Braem et al., 2012; Corthay et al., 2000).

In fact, exercise triggered a pro-tenogenic profile, as revealed by an increase in *Scx*, *Mkx* and *Tnmd* expression in the joint samples. No clear evidence of a chondrogenic response was noticed. Since Sox9 (together with Sox5 and Sox6) is a known enhancer for both *Col2a1* and *Acan* gene promoters (Han and Lefebvre, 2008), our results showing a different response for these 3 genes were startling. Overall, no process resembling endochondral ossification was observed, neither a reorientation of fibro-chondrocytes nor a metaplasia of tendon cells into an osteo-chondrocyte phenotype. Voluntary wheel running seemed to counteract any opportunity for ectopic ossification and rather favored tendon anabolism. Our in vivo results confirm previous data on the effect of stretch on cultured tendon cells, which included the stimulation of the tenogenic transcription factors *Scx* and *Mhk*, and even *Egr1*, the nuclear translocation of Scx, and the increase in *Tnmd* gene and protein expression (Xu et al., 2021; Zhang and Wang, 2013; Yang and Richardson, 2021; Nam et al., 2015; Kubo et al., 2020). Overexpression of Scx and Tnmd has also been demonstrated in the Achilles tendon of mice subjected to several weeks of treadmill training, particularly in epitenon regions (Mendias et al., 2012). It is interesting to note that, in order to promote tenogenic differentiation, mechanical stimulation must remain moderate, as too high a stress could induce *Runx2*, *Sox9* or metalloproteases in these cells (Zhang and Wang, 2013; Kubo et al., 2020). Further investigations are needed to examine whether a longer duration of voluntary running would still favor tendon anabolism or switch the balance toward ectopic ossification process.

As gene expression of *Bmp2*, *Bmp4*, and *Id1* was also reduced in joint cells in response to exercise, we suggest that an off-regulation of BMP signaling could be partly responsible for their pro-tenogenic mechanosensitive response. In particular, regulation of *Tnmd* gene transcription seems to be influenced either by the BMP/Smad/Id1 pathway in the absence of exercise, or by Mkx when cells are mechanically stimulated. These preliminary results need to be further explored, but the putative involvement of BMP signaling makes sense. BMPs expression in both chondrocytes and tendon-derived cells is particularly sensitive to mechanical stimulation (Briolay et al., 2020; Rui et al., 2011). In addition, these cells are especially receptive to BMP signaling in ossifying pathologies, such as calcific tendinopathy, heterotopic ossification in ligaments and tendons, or SpA (Lui et al., 2009; Yonemori et al., 1997; Yang et al., 2015; Dai et al., 2020; Jo et al., 2023). BMP signaling, mechanotransduction pathways and ossification process seem to be closely linked in these pathologies (Yamaguchi et al., 2023).

Lastly, it is important to recall that our protocol was tested with regular healthy mice, which displayed no sign of inflammation. Of course, different outcomes are expected using mice with acute inflammation in arthritic models or low-grade inflammation in models associated with metabolic syndrome, for example (Cambré et al., 2018; Ma et al., 2010).

## 5 Conclusion

We believe that our experimental design could be useful to study the role of mechanical stimulation specifically in the joint, in various conditions. The lack of effect on the long bones makes it possible to focus on the impact of exercise on the enthesis homeostasis, and to further explore the role of mechanical stimulation in the pathophysiology of various enthesopathies.

The following is the supplementary data related to this article.

Suppl. Fig. 1 Hematoxylin-eosin-saffron (HES) staining of the zone of interest within the ankle for the “joint samples” analysis. The V-shaped lines indicate the tissues collected for total RNA isolation. T: tibia, C: calcaneus, AT: Achilles tendon, circle: main enthesis of the ankle, scale bar = 1000 μm. Suppl. Fig. 1

## CRediT authorship contribution statement

**Anne Briolay:** Data curation, Formal analysis, Investigation, Writing – review & editing. **François Duboeuf:** Investigation, Writing – review & editing. **Séverine Delplace:** Investigation, Writing – review & editing. **Leyre Brizuela:** Formal analysis, Writing – review & editing. **Olivier Peyruchaud:** Formal analysis, Writing – review & editing. **David Magne:** Conceptualization, Writing – review & editing. **Carole Bougault:** Conceptualization, Data curation, Formal analysis, Funding acquisition, Investigation, Project administration, Writing – original draft, Writing – review & editing.

## Declaration of competing interest

All the authors declare that they have no known competing financial interests or personal relationships that could have appeared to influence the work reported in this paper.

## Data Availability

Data will be made available on request.

## References

[bb0005] Aghajanian P., Mohan S. (2018). The art of building bone: emerging role of chondrocyte-to-osteoblast transdifferentiation in endochondral ossification. Bone Res.

[bb0010] Benjamin M., Rufai A., Ralphs J.R. (2000). The mechanism of formation of bony spurs (enthesophytes) in the achilles tendon. Arthritis Rheum..

[bb0015] Benjamin M., Toumi H., Ralphs J.R., Bydder G., Best T.M., Milz S. (2006). Where tendons and ligaments meet bone: attachment sites (‘entheses’) in relation to exercise and/or mechanical load. J. Anat..

[bb0020] Bonnet N., Standley K.N., Bianchi E.N., Stadelmann V., Foti M., Conway S.J., Ferrari S.L. (2009). The matricellular protein periostin is required for sost inhibition and the anabolic response to mechanical loading and physical activity. J. Biol. Chem..

[bb0025] Braem K., Deroose C.M., Luyten F.P., Lories R.J. (2012). Inhibition of inflammation but not ankylosis by glucocorticoids in mice: further evidence for the entheseal stress hypothesis. Arthritis Res. Ther..

[bb0030] Briolay A., El Jamal A., Arnolfo P., Le Goff B., Blanchard F., Magne D., Bougault C. (2020). Enhanced BMP-2/BMP-4 ratio in patients with peripheral spondyloarthritis and in cytokine- and stretch-stimulated mouse chondrocytes. Arthritis Res. Ther..

[bb0035] Cambré I., Gaublomme D., Burssens A., Jacques P., Schryvers N., De Muynck A., Meuris L., Lambrecht S., Carter S., de Bleser P., Saeys Y., Van Hoorebeke L., Kollias G., Mack M., Simoens P., Lories R., Callewaert N., Schett G., Elewaut D. (2018). Mechanical strain determines the site-specific localization of inflammation and tissue damage in arthritis. Nat. Commun..

[bb0040] Castro A.A., Karakostis F.A., Copes L.E., McClendon H.E., Trivedi A.P., Schwartz N.E., Garland T. (2021). Effects of selective breeding for voluntary exercise, chronic exercise, and their interaction on muscle attachment site morphology in house mice. J. Anat..

[bb0045] Chan W.C.W., Tan Z., M.K.T. To, Chan D. (2021). Regulation and role of transcription factors in osteogenesis. Int. J. Mol. Sci..

[bb0050] Chang S.H., Yasui T., Taketomi S., Matsumoto T., Kim-Kaneyama J.R., Omiya T., Hosaka Y., Inui H., Omata Y., Yamagami R., Mori D., Yano F., Chung U., Tanaka S., Saito T. (2016). Comparison of mouse and human ankles and establishment of mouse ankle osteoarthritis models by surgically-induced instability. Osteoarthr. Cartil..

[bb0055] Chen H., Li S., Xiao H., Wu B., Zhou L., Hu J., Lu H. (2021). Effect of exercise intensity on the healing of the bone-tendon interface: a mouse rotator cuff injury model study. Am. J. Sports Med..

[bb0060] Corthay A., Hansson A.S., Holmdahl R. (2000). T lymphocytes are not required for the spontaneous development of entheseal ossification leading to marginal ankylosis in the DBA/1 mouse. Arthritis Rheum..

[bb0065] Dai G., Li Y., Liu J., Zhang C., Chen M., Lu P., Rui Y. (2020). Higher BMP expression in tendon stem/progenitor cells contributes to the increased heterotopic ossification in Achilles tendon with aging. Front. Cell Dev. Biol..

[bb0070] De Palma L., Marinelli M., Memè L., Pavan M. (2004). Immunohistochemistry of the enthesis organ of the human Achilles tendon. Foot Ankle Int..

[bb0075] El Jamal A., Briolay A., Mebarek S., Le Goff B., Blanchard F., Magne D., Brizuela L., Bougault C. (2019). Cytokine-induced and stretch-induced sphingosine 1-phosphate production by enthesis cells could favor abnormal ossification in spondyloarthritis. J. Bone Miner. Res..

[bb0085] Fabre S., Molto A., Dadoun S., Rein C., Hudry C., Kreis S., Fautrel B., Pertuiset E., Gossec L. (2016). Physical activity in patients with axial spondyloarthritis: a cross-sectional study of 203 patients. Rheumatol. Int..

[bb0090] Flammier S., Peyruchaud O., Bourguillault F., Duboeuf F., Davignon J.L., Norman D.D., Isaac S., Marotte H., Tigyi G., Machuca-Gayet I., Coury F. (2019). Osteoclast-derived autotaxin, a distinguishing factor for inflammatory bone loss. Arthritis Rheumatol..

[bb0095] Gerbaix M., Vico L., Ferrari S.L., Bonnet N. (2015). Periostin expression contributes to cortical bone loss during unloading. Bone.

[bb0100] Guilak F. (2011). Biomechanical factors in osteoarthritis. Best Pract. Res. Clin. Rheumatol..

[bb0105] Han Y., Lefebvre V.R. (2008). L-Sox5 and Sox6 drive expression of the aggrecan gene in cartilage by securing binding of Sox9 to a far-upstream enhancer. Mol. Cell. Biol..

[bb0110] Hughes J.M., Petit M.A. (2010). Biological underpinnings of Frost’s mechanostat thresholds: the important role of osteocytes. J. Musculoskelet. Neuronal Interact..

[bb0115] Isaksson H., Tolvanen V., Finnila M.A., Iivarinen J., Tuukkanen J., Seppanen K., Arokoski J.P., Brama P.A., Jurvelin J.S., Helminen H.J. (2009). Physical exercise improves properties of bone and its collagen network in growing and maturing mice. Calcif. Tissue Int..

[bb0120] Jacques P., McGonagle D. (2014). The role of mechanical stress in the pathogenesis of spondyloarthritis and how to combat it. Best Pract. Res. Clin. Rheumatol..

[bb0125] Jacques P., Lambrecht S., Verheugen E., Pauwels E., Kollias G., Armaka M., Verhoye M., Van der Linden A., Achten R., Lories R.J., Elewaut D. (2013). Proof of concept: enthesitis and new bone formation in spondyloarthritis are driven by mechanical strain and stromal cells. Ann. Rheum. Dis..

[bb0130] Jo S., Lee S.H., Jeon C., Jo H.R., Ko E., Whangbo M., Kim T.J., Park Y.S., Kim T.H. (2023). Elevated BMPR2 expression amplifies osteoblast differentiation in ankylosing spondylitis. J Rheum Dis.

[bb0135] Kan C., Chen L., Hu Y., Ding N., Lu H., Li Y., Kessler J.A., Kan L. (2018). Conserved signaling pathways underlying heterotopic ossification. Bone.

[bb0140] Kubo Y., Hoffmann B., Goltz K., Schnakenberg U., Jahr H., Merkel R., Schulze-Tanzil G., Pufe T., Tohidnezhad M. (2020). Different frequency of cyclic tensile strain relates to anabolic/catabolic conditions consistent with immunohistochemical staining intensity in tenocytes. Int. J. Mol. Sci..

[bb0145] Lencel P., Delplace S., Pilet P., Leterme D., Miellot F., Sourice S., Caudrillier A., Hardouin P., Guicheux J., Magne D. (2011). Cell-specific effects of TNF-α and IL-1β on alkaline phosphatase: implication for syndesmophyte formation and vascular calcification. Lab. Invest..

[bb0150] Lerman I., Harrison B.C., Freeman K., Hewett T.E., Allen D.L., Robbins J., Leinwand L.A. (2002). Genetic variability in forced and voluntary endurance exercise performance in seven inbred mouse strains. J. Appl. Physiol.(1985).

[bb0155] Lewis T.C., Prywes R. (2013). Serum regulation of Id1 expression by a BMP pathway and BMP responsive element. Biochim. Biophys. Acta.

[bb0160] Liu H., Zhu S., Zhang C., Lu P., Hu J., Yin Z., Ma Y., Chen X., OuYang H. (2014). Crucial transcription factors in tendon development and differentiation: their potential for tendon regeneration. Cell Tissue Res..

[bb0165] Lories R.J., Schett G. (2012). Pathophysiology of new bone formation and ankylosis in spondyloarthritis. Rheum. Dis. Clin. North Am..

[bb0170] Lu H., Li S., Zhang T., Wang Z., Chen C., Chen H., Xiao H., Wang L., Chen Y., Tang Y., Xie S., Wu B., Hu J. (2021). Treadmill running initiation times and bone-tendon interface repair in a murine rotator cuff repair model. J. Orthop. Res..

[bb0175] Lui P.P., Chan L.S., Cheuk Y.C., Lee Y.W., Chan K.M. (2009). Expression of bone morphogenetic protein-2 in the chondrogenic and ossifying sites of calcific tendinopathy and traumatic tendon injury rat models. J. Orthop. Surg. Res..

[bb0180] Ma H., Torvinen S., Silvennoinen M., Rinnankoski-Tuikka R., Kainulainen H., Morko J., Peng Z., Kujala U.M., Rahkila P., Suominen H. (2010). Effects of diet-induced obesity and voluntary wheel running on bone properties in young male C57BL/6J mice. Calcif. Tissue Int..

[bb0185] Magne D., Bougault C. (2015). What understanding tendon cell differentiation can teach us about pathological tendon ossification. Histol. Histopathol..

[bb0190] Martin J.A., Mehr D., Pardubsky P.D., Buckwalter J.A. (2003). The role of tenascin-C in adaptation of tendons to compressive loading. Biorheology.

[bb0195] Mendias C.L., Gumucio J.P., Bakhurin K.I., Lynch E.B., Brooks S.V. (2012). Physiological loading of tendons induces scleraxis expression in epitenon fibroblasts. J. Orthop. Res..

[bb0200] Nam H.Y., Pingguan-Murphy B., Amir Abbas A., Mahmood Merican A., Kamarul T. (2015). The proliferation and tenogenic differentiation potential of bone marrow-derived mesenchymal stromal cell are influenced by specific uniaxial cyclic tensile loading conditions. Biomech. Model. Mechanobiol..

[bb0205] Neerinckx B., Lories R. (2017). Mechanisms, impact and prevention of pathological bone regeneration in spondyloarthritis. Curr. Opin. Rheumatol..

[bb0210] Nookaew I., Xiong J., Onal M., Bustamante-Gomez C., Wanchai V., Fu Q., Kim H.N., Almeida M., O’Brien C.A. (2024). Refining the identity of mesenchymal cell types associated with murine periosteal and endosteal bone. J. Biol. Chem..

[bb0215] Nordling C., Karlsson-Parra A., Jansson L., Holmdahl R., Klareskog L. (1992). Characterization of a spontaneously occurring arthritis in male DBA/1 mice. Arthritis Rheum..

[bb0220] Portier H., Benaitreau D., Pallu S. (2020). Does physical exercise always improve bone quality in rats?. Life (Basel).

[bb0225] Rui Y.F., Lui P.P., Ni M., Chan L.S., Lee Y.W., Chan K.M. (2011). Mechanical loading increased BMP-2 expression which promoted osteogenic differentiation of tendon-derived stem cells. J. Orthop. Res..

[bb0230] Sapir-Koren R., Livshits G. (2014). Osteocyte control of bone remodeling: is sclerostin a key molecular coordinator of the balanced bone resorption–formation cycles?. Osteoporos. Int..

[bb0235] Schlecht S.H., Ramcharan M.A., Yang Y., Smith L.M., Bigelow E.M., Nolan B.T., Moss D.E., Devlin M.J., Jepsen K.J. (2018). Differential adaptive response of growing bones from two female inbred mouse strains to voluntary cage-wheel running. JBMR Plus.

[bb0240] Seward T., Harfmann B.D., Esser K.A., Schroder E.A. (2018). Reinventing the wheel: comparison of two wheel cage styles for assessing mouse voluntary running activity. J. Appl. Physiol. (1985).

[bb0245] Shukunami C., Takimoto A., Nishizaki Y., Yoshimoto Y., Tanaka S., Miura S., Watanabe H., Sakuma T., Yamamoto T., Kondoh G., Hiraki Y. (2018). Scleraxis is a transcriptional activator that regulates the expression of Tenomodulin, a marker of mature tenocytes and ligamentocytes. Sci. Rep..

[bb0250] Silvennoinen M., Rantalainen T., Kainulainen H. (2014). Validation of a method to measure total spontaneous physical activity of sedentary and voluntary running mice. J. Neurosci. Methods.

[bb0255] Tits A., Plougonven E., Blouin S., Hartmann M.A., Kaux J.-F., Drion P., Fernandez J., Van Lenthe G.H., Ruffoni D. (2021). Local anisotropy in mineralized fibrocartilage and subchondral bone beneath the tendon-bone interface. Sci. Rep..

[bb0260] Waggett A.D., Ralphs J.R., Kwan A.P., Woodnutt D., Benjamin M. (1998). Characterization of collagens and proteoglycans at the insertion of the human Achilles tendon. Matrix Biol..

[bb0265] Xu P., Deng B., Zhang B., Luo Q., Song G. (2021). Stretch-induced tenomodulin expression promotes tenocyte migration via F-actin and chromatin remodeling. Int. J. Mol. Sci..

[bb0270] Yamaguchi H., Li M., Kitami M., Swaminathan S., Mishina Y., Komatsu Y. (2023). Enhanced BMP signaling in cathepsin K-positive tendon progenitors induces heterotopic ossification. Biochem. Biophys. Res. Commun..

[bb0275] Yang F., Richardson D.W. (2021). Comparative analysis of tenogenic gene expression in tenocyte-derived induced pluripotent stem cells and bone marrow-derived mesenchymal stem cells in response to biochemical and biomechanical stimuli. Stem Cells Int..

[bb0280] Yang M., Yuan H., Miao M., Xu W. (2015). The osteogenic potential of ligament fibroblasts is greater in ankylosing spondylitis patients than in patients with osteoarthritis. Z. Rheumatol..

[bb0285] Yonemori K., Imamura T., Ishidou Y., Okano T., Matsunaga S., Yoshida H., Kato M., Sampath T.K., Miyazono K., ten Dijke P., Sakou T. (1997). Bone morphogenetic protein receptors and activin receptors are highly expressed in ossified ligament tissues of patients with ossification of the posterior longitudinal ligament. Am. J. Pathol..

[bb0290] Zhang J., Wang J.H.C. (2013). The effects of mechanical loading on tendons - an in vivo and in vitro model study. PloS One.

[bb0295] Zhang J., Valverde P., Zhu X., Murray D., Wu Y., Yu L., Jiang H., Dard M.M., Huang J., Xu Z., Tu Q., Chen J. (2017). Exercise-induced irisin in bone and systemic irisin administration reveal new regulatory mechanisms of bone metabolism. Bone Res.

