# Supplementary material for: Voluntary exercise in mice triggers an anti-osteogenic and pro-tenogenic response in the ankle joint without affecting long bones
Source: Bone Rep. 2024 Oct 15;23:101810. doi: 10.1016/j.bonr.2024.101810 (PMC11530850; doi:10.1016/j.bonr.2024.101810)
Supplement: Suppl. Fig. 1 — Hematoxylin-eosin-saffron (HES) staining of the zone of interest within the ankle for the “joint samples” analysis. The V-shaped lines indicate the tissues collected for total RNA isolation. T: tibia, C: calcaneus, AT: Achilles tendon, circle: main enthesis of the ankle, scale bar = 1000 μm. [file mmc1.docx]

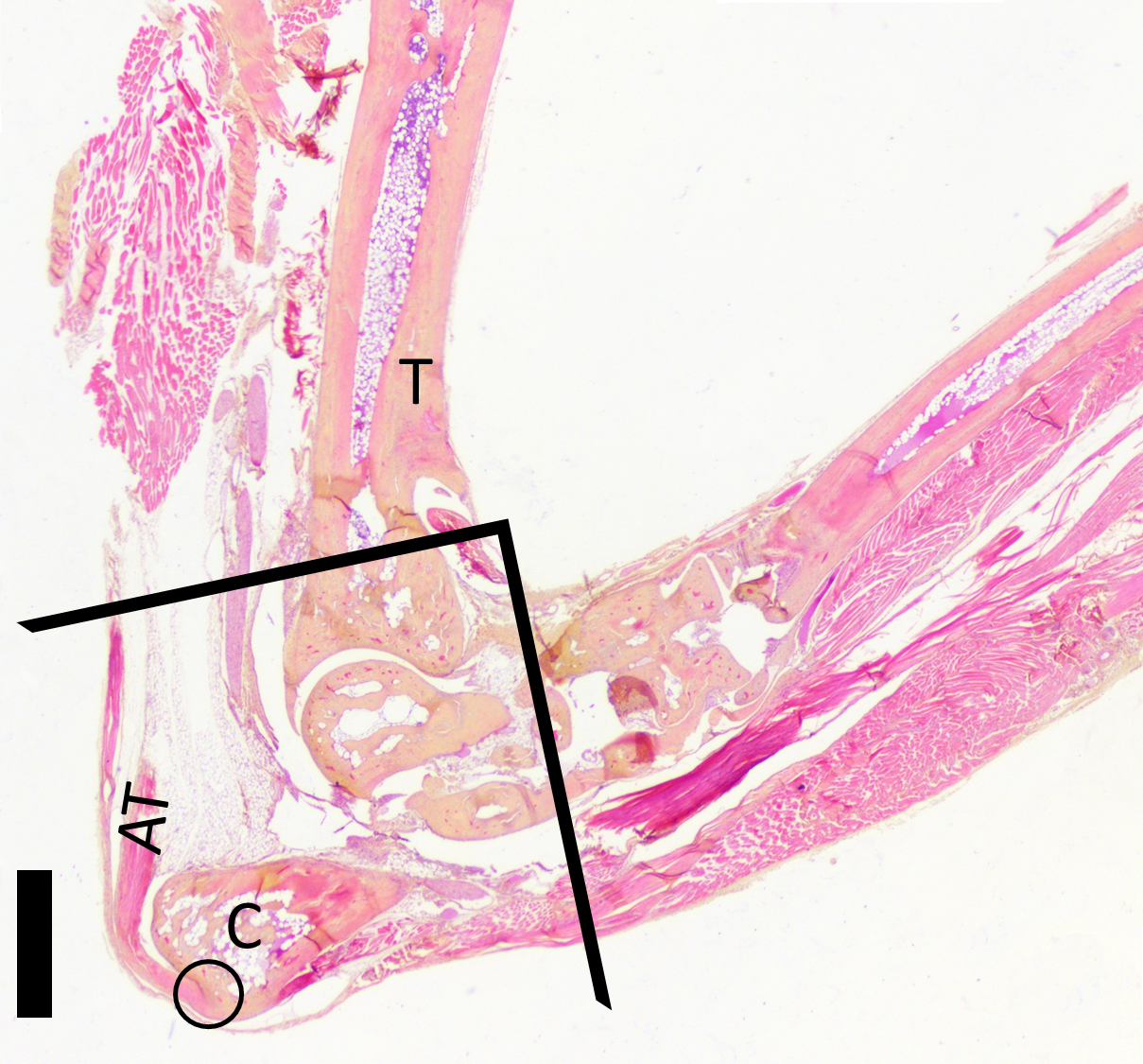


**Suppl. Fig. 1:** Hematoxylin-eosin-saffron (HES) staining of the zone of interest within the ankle for the “joint samples” analysis. The V-shaped lines indicate the tissues collected for total RNA isolation. T: tibia, C: calcaneus, AT: Achilles tendon, circle: main enthesis of the ankle, scale bar = 1000 µm.
